# Predicting Non-Alcoholic Fatty Liver Disease Progression and Immune Deregulations by Specific Gene Expression Patterns

**DOI:** 10.3389/fimmu.2020.609900

**Published:** 2021-01-26

**Authors:** Fanhong Zeng, Yue Zhang, Xu Han, Min Zeng, Yi Gao, Jun Weng

**Affiliations:** ^1^ Department of Hepatobiliary Surgery II, Guangdong Provincial Research Center for Artificial Organ and Tissue Engineering, Guangzhou Clinical Research and Transformation Center for Artificial Liver, Institute of Regenerative Medicine, ZhuJiang Hospital, Southern Medical University, Guangzhou, China; ^2^ State Key Laboratory of Organ Failure Research, Southern Medical University, Guangzhou, China

**Keywords:** non-alcoholic fatty liver disease, inflammation, signature classifiers, immune microenvironment, drug sensitivity

## Abstract

Non-alcoholic fatty liver disease (NAFLD) is the most common liver disease worldwide with rising rates in parallel to obesity, type 2 diabetes, and metabolic syndrome. NAFLD includes pathologies ranging from simple steatosis (NAFL) to non-alcoholic steatohepatitis and cirrhosis (NASH), which may eventually develop into hepatocellular carcinoma (HCC). Mechanically, lipids accumulation and insulin resistance act as the first hit, inflammation and fibrosis serve as the second hit. Currently, the diagnosis of NAFLD mainly depends on pathology examination and medical imaging, whereas proper gene signature classifiers are necessary for the evaluation of disease status. Here, we developed three signature classifiers to distinguish different NAFLD disease states (NAFL and NASH). Moreover, we found that B cells, DCs, and MAIT cells are key deregulated immune cells in NAFLD, which are associated with NAFLD and NAFLD-HCC progression. Meanwhile, AKR1B10 and SPP1 are closely related to the above three immune cell infiltrations and immunosuppressive cytokines expressions in NAFLD and NAFLD-HCC. Subsequently, we screened out AKR1B10 and SPP1 sensitive molecules TGX-221, which may provide a possible therapy for NAFLD and NAFLD-HCC.

## Introduction

During the past century, Non-alcoholic Fatty Liver disease (NAFLD) has become one of the most important causes of liver disease and it may become the leading cause of end-stage liver disease in the next few decades (1–3). Currently, the global prevalence of NAFLD is estimated to be 24%, the highest rates are reported in South America (31%) and the lowest in Africa (14%) (4). NAFLD is strongly associated with metabolic syndromes, such as obesity, type 2 diabetes mellitus, dyslipidemia, and hypertension (5, 6). Abnormal lifestyle (Caloric excess and sedentary lifestyle) is the major cause of NAFLD. As the rates of obesity continue to rise, the prevalence of NAFLD is constantly increasing in the past decade from 15% in 2005 to 25% in 2010 worldwide (4, 5).

NAFLD is considered as a complex disease trait, and interactions between the environment and a susceptible polygenic host background determine disease phenotype and influence progression (4). Lots of genome-wide association and large candidate gene studies indicated that PNPLA3, TM6SF2, MBOAT7, LYPLAL1, APOB, and GCKR variants were important genetic and epigenetic modifiers of NAFLD progression in specific populations and races (7, 8). However, the universal mechanism of NAFLD occurrence and progression remains elusive (6). So far, the most recognized theory is the ‘two-hit’ theory, namely, abnormal lipid metabolism and inflammatory storm (9, 10). The first-hit is abnormal liver lipid metabolism, resulting in excessive lipid influx or/and decreased lipid clearance. In this progress, steatosis may be reversible and does not necessarily cause permanent liver damage (11). The second-hit is the inflammatory storm process, which may be caused by oxidative stress, lipid peroxidation and cytokine action. Although the second-hit occurs less frequently, it is more toxic and irreversible, as lobular inflammation directly leads to ballooning degeneration and perisinusoidal fibrosis, which promotes apoptosis and liver cell death, and finally leads to scarring and progression to non-alcoholic steatohepatitis (NASH) (10). Therefore, understandings about two ‘hits’ molecular mechanisms and prognostic biomarkers are essential to NAFLD prevention and treatment.

In our study, we developed three classifiers to classify different NAFLD states, exhibiting NAFLD associated genes to reflect NAFLD immune microenvironment deregulation and progression, and predicting potential therapeutic targets and drugs.

## Materials and Methods

### Data Processing

Forty-four patients in GSE33814, 73 patients in GSE48452 and 63 patients in GSE89632 were downloaded from Gene Expression Omnibus (GEO), 45 patients in E-MEXP-3291 were from the ArrayExpress database. We used the “SVA” package in R for batch correction (12). HCC data were contained from The Cancer Genome Atlas database (TCGA), including 374 HCC samples and 50 normal samples.

The immunohistochemical pictures were acquired from the HPA database (https://www.proteinatlas.org/), the survival analysis from Gepia (http://gepia.cancer-pku.cn/) and co-expression network from cBioPortal database. ImmuCellAI (http://bioinfo.life.hust.edu.cn/ImmuCellAI#!/) was used to analyze the patient’s immune status (13). Besides, drug-sensitive data were collected from GDSC database in GSCALite (http://bioinfo.life.hust.edu.cn/web/GSCALite/).

### Diagnostic Methods to Diagnose Different States of NAFLD

The diagnoses of all samples (E-MEXP-3291, GSE33814, GSE48452, and GSE89632) used were histologically validated by a board-certified pathologist before molecular analysis. For histological analysis, hematoxylin and eosin (H&E) and chromotrope aniline blue (CAB) stained sections were used. Histological slides were diagnosed using criteria from a scoring system for human NAFLD (14). Besides, in the TCGA database, HCC samples used were histologically validated by a board-certified pathologist before molecular analysis. The standards were defined by the American Association for the Study of Liver Diseases (AASLD) (15). Information on donors, including age and gender, can be seen in **Table 1**.

**Table 1:** Clinical Characteristics of E-MEXP-3291, GSE33814, GSE48452, GSE89632 and TCGA-HCC.

E-MEXP-3291		Normal (n=19)	NAFL(n=10)	NASH(n=16)	*p*
Gender	Male	9	5	3	>0.05
	Female	10	4	12	
Age (years)		43.26 (16-70)	44.66 (16-66)	55.93 (41-68)	>0.05
GSE33814		Normal(n=10)	NAFL(n=14)	NASH (n=8)	*p*
Gender	Male	5	8	6	0.43
	Female	5	6	2	
Age (years)		51 (25–73)	61.5 (37–78)	55 (46–72)	0.37
Ballooning (0:1:2)		10:0:0	14:0:0	0:4:4	
Fibrosis (0: 1:2:3:4)		9: 1:0:0:0	9: 4:1:0:0	0: 0:0:2:6	
Inflammation (0:1:2:3)		6:3:1:0	1:7:5:1	1:3:3:1	
Steatosis (0:1:2:3)		10:0:0:0	0:10:3:1	3:1:4:0	
Matteoni (points)		n/a	2 (1–2)	3 (0–4)	
BMI		22.8(18.3-30.1)	25.5(19.8-39.2)	31(21.4-30.8)	
GSE48452		Normal (n=41)	NAFL (n=14)	NASH (n=18)	*p*
Gender	Male	7	4	4	>0.05
	Female	34	10	14	
Age (years)		47.56 (23-80)	41.60 (24-65)	45.48 (30-58)	>0.05
Steatosis (0-30%:30-50%:>50%)		41:00:00	5:05:04	0:3:15	
Inflammation (0:1:2-3)		37:03:00	12:02:00	0:9:9	
Fibrosis (0:1:2-4)		33:05:01	10:04:00	3:11:4	
NAS (0-2:3-4:5-8)		41:00:00	11:03:00	0:3:15	
BMI		35.36 (17-55)	48.28(40-60)	45.97(24-70)	
GSE89632		Normal (n=24)	NAFL (n=20)	NASH (n=19)	*p*
Gender	Male	11	14	9	>0.05
	Female	13	6	10	
Age (years)		37.21(22-58)	44.7(30-60)	43.47(23-68)	>0.05
Ballooning (0:1:2)		17:0:0	20:0:0	0:13:6	
Steatosis (0-30%:30-50%:>50%)		17:0:0	9:7:4	5:7:7	
Inflammation (0:1:2-3)		7:0:0	19:0:0	0:11:8	
Fibrosis (0:1:2-4)		10:6:0	17:3:0	4:5:10	
NAS (0-2:3-4:5-8)		7:0:0	16:3:0	0:9:10	
BMI		25.88(18-33)	28.78(22-41)	31.77(23-49)	
TCGA Database		Category		Number of patients	
Gender		Female		122	
		Male		255	
Age(years)		<65		224	
		≥65		153	
Grade		I		55	
		II		180	
		III		124	
		IV		13	
Stage		1		175	
		2		87	
		3		86	
		4		5	
Status		Alive		249	
		Dead		128	

### Differential Analysis, GO\KEGG Analysis, and Gene Set Enrichment Analyses (GSEA)

The genes differentially expressed (DEGs) were calculated and labeled using the “Limma” package. Subsequently, DEGs were analyzed by gene ontology (GO) and Kyoto Encyclopedia of Genes and Genomes (KEGG) pathway analyses. GO analysis consists of three parts, including molecular function (MF), biological process (BP), and cell component (CC). We determined that results are statistically significant at a level of less than 0.05 using a p-value. The GSEA was performed by GSEA software (version 4.0.3). We utilized it to detect the difference in the set of genes expressed between the high-risk and low-risk group in the enrichment of the MSigDB Collection (h.all.v7.1.symbols.gmt). For each analysis, gene set permutations were performed 1000 times.

### Weighted Gene Co-Expression Network Analysis (WGCNA)

To find modules highly correlated with NAFLD, WGCNA was performed using the WGCNA R package and carried out on all genes (16, 17). The Pearson correlation coefficient was used to establish an unsupervised co-expression relationship based on the connection strength adjacency matrix for gene pairs. This matrix was increased to β = 7 based on the scale-free topology criterion. Then the topological overlap matrix was used to analyze the adjacency matrix of clustering GC patient gene expression data. Finally, the dynamic tree cut algorithm was applied to the dendrogram for module identification with the mini-size of module gene numbers set as 50 and a cut height of 0.9. In the module-trait analysis, GS value>0.3 and MM value>0.55 were defined as a threshold. The WGCNA algorithm was described in detail by Zhang Bin et al. (18).

### Protein-Protein Interaction (PPI) Network Construction

STRING database (https://cytoscape.org/) was used to construct a protein-protein interaction network (PPI). We hid disconnected nodes in the network and set the minimum required interaction score to 0.4. Later, the Cytoscape software (Version 3.7.1) was applied to visualize the PPI network. The data were imported into CytoHubba plugin, which helped to identify key genes through five different calculation methods, namely, EPC, MCC, DMNC, MNC, and Degree (19). Then, the processed data were imported into another plugin, MCODE, which helped to identify different clusters (20).

### The LASSO Logistic Regression

The LASSO logistic regression model analysis used the “glmnet” package in R (12). We extracted the expression of 9 core genes for each NAFLD sample. While selecting the optimal features of high-dimensional data, the LASSO method that prevents overfitting with strong predictive values and low mutual correlation was used (21). Principal component analysis (PCA) was performed before/after feature selection using the expression profiles of 9 core genes. The cores genes, as optimal genes, which were identified with non-zero regression coefficients, were used to establish an mRNA-based signature classifier for predicting different NAFLD states. A classifier index for each sample was created with the following formula: index = Exp gene1 *Coef1 + Exp gene2 *Coef2 + Exp gene3*Coef3+ …

The efficiency of the classifier was assessed by mean squared errors (MSE), accuracy, sensitivity (Se), specificity (Sp), positive predictive value (PPV), negative predictive value (NPV), and area under the receiver operating characteristic (ROC) curve. These ROC curves were drawn and compared using the “pROC” package in R (22).

## Result

### Identification of DEGs in NAFLD Patients

**Figure 1:**
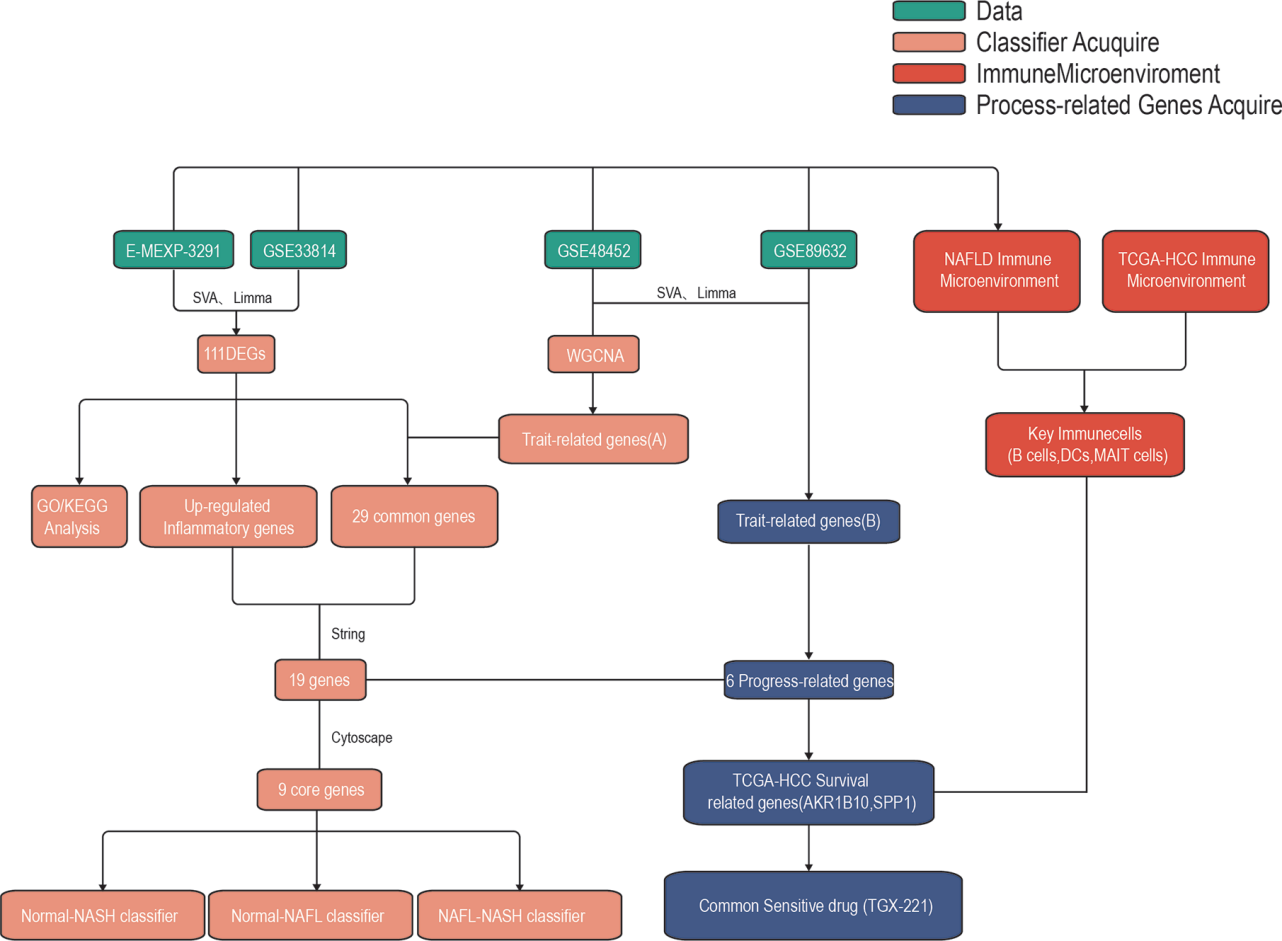
The workflow of the present study.

**Figure 2:**
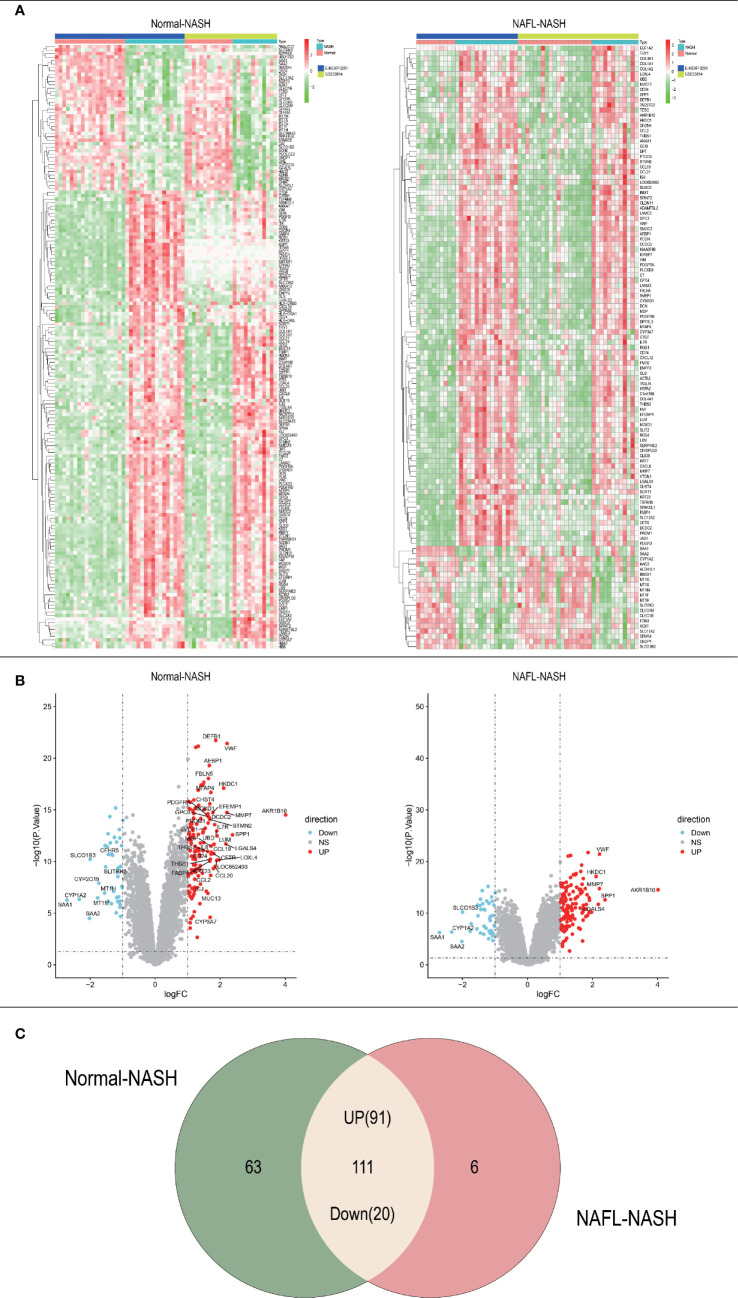
Acquisition of differentially expressed genes (DEGs). **(A)** Heatmap of Normal-NASH group and NAFL-NASH group; **(B)** Volcano map of Normal-NASH group and NAFL-NASH group; **(C)** Venn diagram between Normal-NASH group and NAFL-NASH group.

The whole research design was illustrated in **Figure 1**. Firstly, we identified genes which were expressed differently at different stages of NAFLD. The data of NAFLD obtained from GSE33814 and E-MEXP-3291, including 32 normal samples, 29 non-alcoholic fatty liver (NAFL) samples, and 28 NASH samples. 174 DEGs from Normal-NASH group and 117 DEGs from NAFL-NASH group were included in the analysis, which met the screening standard of our study (p<0.05, |log2FC) | >1.0). The expression distributions of these DEGs were displayed in **Figure 2A-B**. Subsequently, we searched for the common genes of these two groups. A total of 111 DEGs were included, consisting of 91 up-regulated and 20 down-regulated DEGs (**Figure 2C**).

### GO and KEGG Pathway Enrichment Analysis and Gene Set Enrichment Analysis (GSEA)

**Figure 3:**
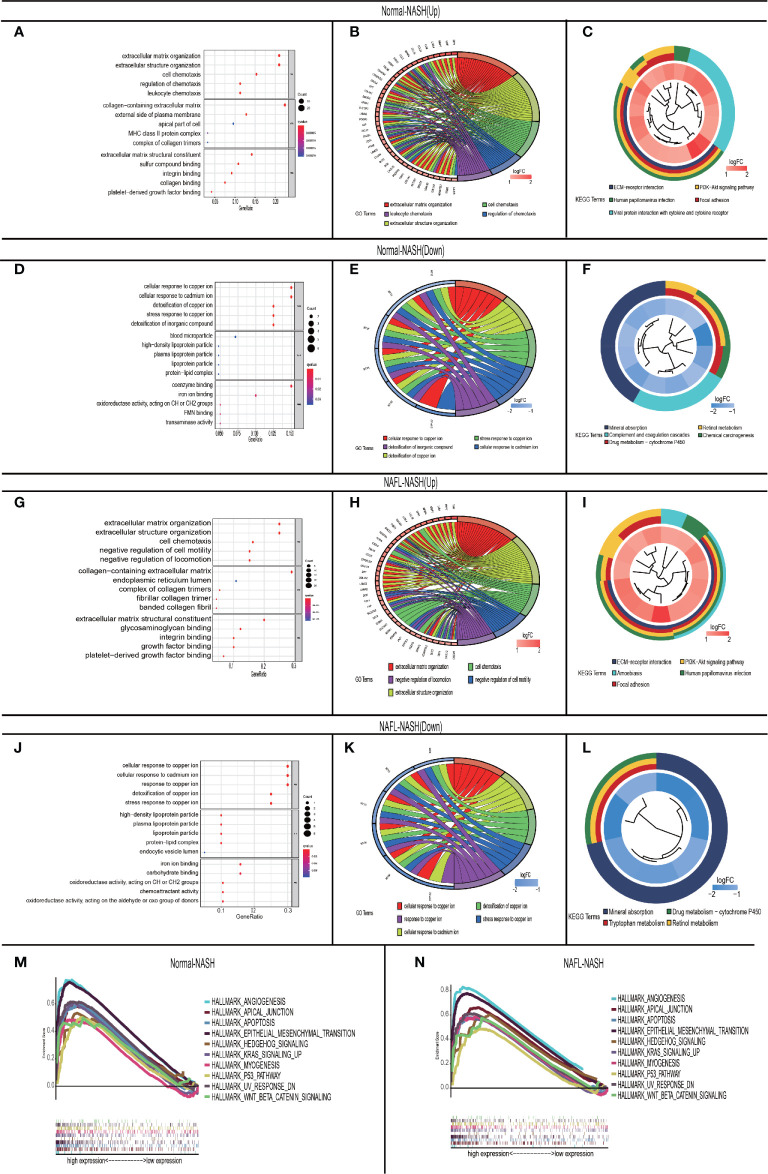
GO\KEGG and GASE in non-alcoholic fatty liver disease. **(A–C)** Up-regulated Normal-NASH group. GO analysis (including molecular function (MF), biological process (BP), and cell component (CC) **(A)**; Top 5 of GO analysis **(B)**; KEGG pathway analysis **(C)**; **(D–F)** Down-regulated Normal-NASH group. GO analysis (including molecular function (MF), biological process (BP), and cell component (CC) **(D)**; Top 5 of GO analysis **(E)**; KEGG pathway analysis **(F)**; **(G-I)** Up-regulated NAFL-NASH group. GO analysis (including molecular function (MF), biological process (BP), and cell component (CC) **(G)**; Top 5 of GO analysis **(H)**; KEGG pathway analysis **(I)**; **(J–L)** Up-regulated NAFL-NASH group. (GO analysis (including molecular function (MF), biological process (BP), and cell component (CC) **(J)**; Top 5 of GO analysis **(K)**; KEGG pathway analysis **(L)**; **(M, N)** Top 10 interactive pathway of GASE in Normal-NASH group **(M)**/NAFL-NASH group **(N)** (GSE33814 and E-MEXP-3291).

To investigate the mechanism of NAFLD progression, we performed GO/KEGG analysis among Normal-NASH and NAFL-NASH group. In the up-regulated group, intersected GO terms were significantly enriched in extracellular matrix organization, extracellular structure organization and cell chemotaxis, which were closely related to tumorigenesis. (**Figures 3A, B, G, H**). And KEGG analysis showed intersected pathways were enriched in cancer-related pathways, such as PI3K−AKT, ECM−receptor interaction, Focal adhesion and Human papillomavirus infection signaling pathway (**Figures 3C, I**). In down-regulated group, the results showed intersected GO terms were significantly enriched in cellular response to copper ion, stress response to copper ion, cellular response to cadmium ion and detoxification of copper ion (**Figures 3D, E, J, K**). Meanwhile, KEGG analysis showed that pathways were enriched in Mineral absorption, Drug metabolism − cytochrome P450 and Retinol metabolism (**Figures 3F, L**).

To further explore the mechanism of NAFLD progression, we performed GSEA which took c7.1 as a reference gene set. The overlapping pathways between Normal-NASH group and NAFL-NASH group were shown in **Table 2**. The results recognized that pathways were significantly associated with tumorigeneses, such as epithelial-mesenchymal transition (EMT), angiogenesis and p53 pathway (**Figures 3M, N**).

**Table 2:** Intersected pathway between Normal-NASH group and NAFL-NASH group *via* GSEA analysis.

NAME	Normal-NASH		Rank	NAFLD-NASH		RANK	TotalRank
	NES	NOM p-value		NES	NOM p-value		
APICAL_JUNCTION	1.7019578	0.006302521	1	1.8813994	≤0.001	1	1
WNT_BETA_CATENIN_SIGNALING	1.5594031	0.033126295	4	1.7948854	0.001945525	2	2
EPITHELIAL_MESENCHYMAL_TRANSITION	1.5607316	0.002070393	3	1.6032957	0.001980198	5	3
MYOGENESIS	1.5583161	0.034836065	5	1.7363774	0.002028398	4	4
P53_PATHWAY	1.6230526	0.008264462	2	1.5556473	0.01980198	7	5
HEDGEHOG_SIGNALING	1.5503728	0.04338843	7	1.7384652	0.005769231	3	6
APOPTOSIS	1.5512007	0.002145923	6	1.5238415	0.01992032	8	7
ANGIOGENESIS	1.4463018	0.029661017	10	1.59207	≤0.001	6	8
KRAS_SIGNALING_UP	1.4812572	0.015118791	8	1.4351604	0.0392562	10	9
UV_RESPONSE_DN	1.4586604	0.029106028	9	1.5220932	0.014084507	9	10

Therefore, the above results indicated that the extracellular matrix may play an extremely important role in the progression of NAFLD.

### WGCNA and Module Identification of NAFLD

**Figure 4:**
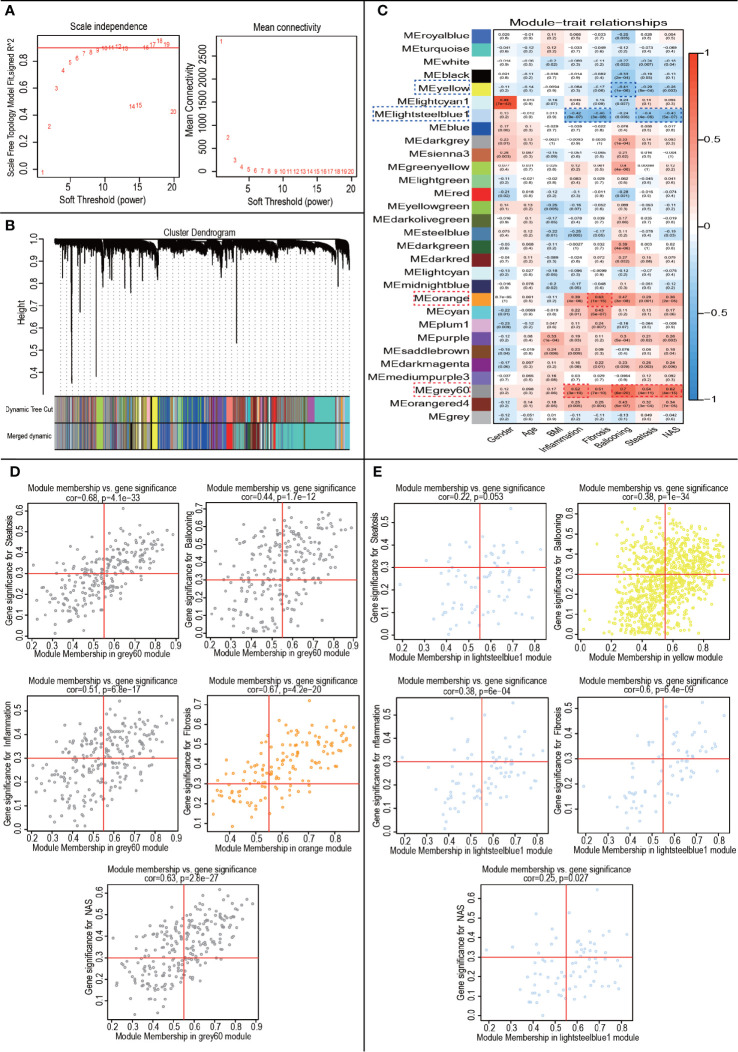
WGCNA to Identify trait-related modules and genes. **(A)** Calculating soft-thresholding power. Left: scale-free fit indices using different soft-thresholding powers (β). Right: mean connectivity using different soft-thresholding powers; **(B)** The dendrogram clustered by Dynamic Tree Cut algorithm; **(C)** The heatmap profiling the correlations between module eigengenes and the clinical characteristics; **(D)** Scatter plot of trait-related modules (Up-regulated); **(E)** Scatter plot of trait-related modules (Down-regulated).

WGCNA was performed to construct co-expressed networks and identify co-expression modules. We obtained RNA expression data and clinical characteristics from GSE48452 and GSE89632. Co-expression analysis was carried out to construct the co-expression network. In our study, the power of β = 7 (scale-free R^2^ = 0.9) was selected as the soft thresholding parameter to ensure a scale-free network (**Figure 4A**). A total of 30 modules were identified through hierarchical clustering (**Figure 4B**). Similar module clustering was constructed by using dynamic hybrid cutting (threshold=0.2). Among the 30 modules, the grey60 module was the highest positive module correlated to ballooning (R^2 =^ 0.7, p=6e^-20^), steatosis (R^2 =^ 0.54, p=4e^-11^), inflammation (R^2 =^ 0.52, p=3e^-10^) and nonalcoholic fatty liver activity score (NAS) (R^2 =^ 0.62, p=4e^-15^). The orange module showed the highest positive correlation with fibrosis (R^2 ^= 0.63, p=1e^-15^). Besides, lightsteelblue1 was highly negatively correlated to fibrosis (R^2 ^= 0.48, p=3e^-8^), steatosis (R^2 ^= 0.4, p=4e^-6^), inflammation (R^2 ^= 0.42, p=9e^-7^) and NAS (R^2 ^= 0.43, p=5e^-7^). The yellow module showed a highly negative correlation with ballooning (R^2 ^= 0.41, p=1e^-6^) (**Figure 4C**). The positive modules were shown in **Figure 4D** and the negative modules in **Figure 4E**.

In the module-trait analysis, GS value > 0.3 and MM value > 0.55 were defined as trait-related genes. Subsequently, we intersected the trait-related genes obtained from WGCNA analysis and 111 DEGs obtained from expression difference analysis and finally obtained 29 trait-expression-related genes.

### Identification of Core Genes and Construction of Protein-Protein Interaction (PPI) Network

**Figure 5:**
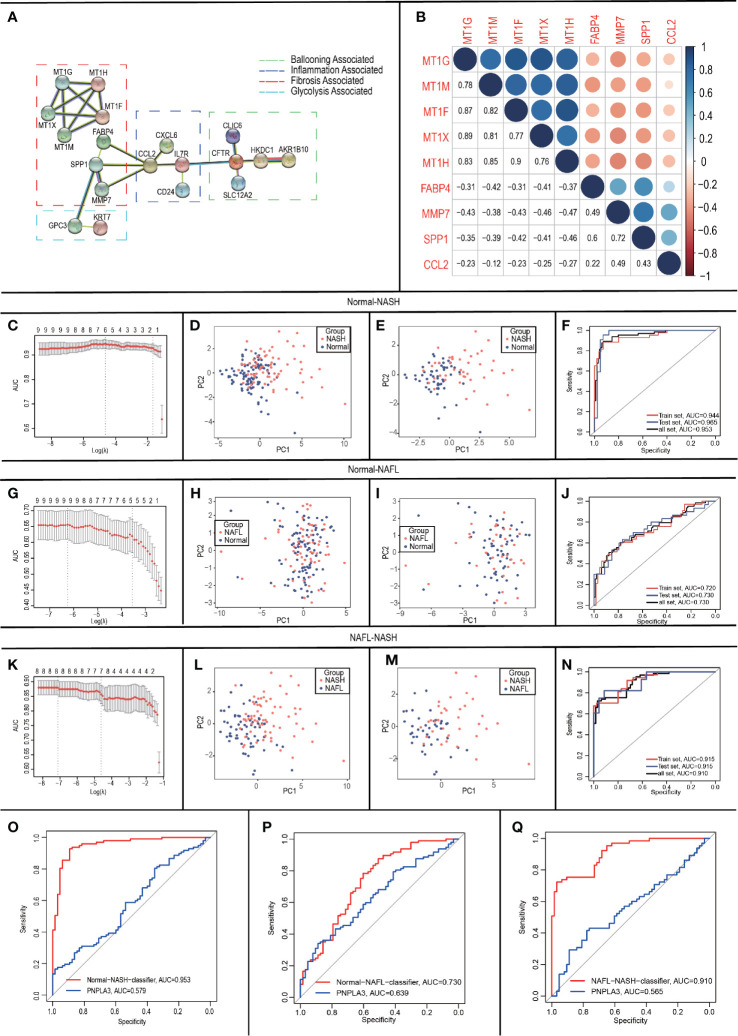
Construction and validation of LASSO logistic regression classifiers. **(A)** Protein-Protein Interaction interactions among 19 DEGs; **(B)** Correlation analysis of nine core genes; **(C-F)** In Normal-NASH group, parameter selection in the LASSO model **(C)**; principal component analysis before **(D)**/after **(E)** parameter selection; Receiver operating characteristic analyses in the training, the testing and the total group **(F)**; **(G-J)** In Normal-NAFL group, parameter selection in the LASSO model **(G)**; principal component analysis before **(H)**/after **(I)** parameter selection; Receiver operating characteristic analyses in the training, the testing and the total group **(J)**; **(K-N)** In NAFL-NASH group, parameter selection in the LASSO model **(K)**; principal component analysis before **(L)**/after **(M)** parameter selection; Receiver operating characteristic analyses in the training, the testing and the total group **(N)**; **(O-Q)** Receiver operating characteristic analyses between classifier and PNPLA3 in Normal-NASH group **(O)**/Normal-NAFL group **(P)**/NAFL-NASH group **(Q)**.

As inflammation plays a central role in NAFLD, we constructed a PPI network with 29 trait-expression-related genes and 5 elevated inflammatory factors obtained from differential analysis. In the STRING database, we defined the removal standard to 0.4 and removed the independent genes. Later, 19 filtered genes were divided into four modules (Ballooning Associated, Inflammation Associated, Fibrosis Associated, and Glycolysis Associated) (**Figure 5A**). The interactive relationship of 19 filtered genes in the whole network was determined using the Cytoscape software (CytoHubba plugin and MCODE plugin). Firstly, the data were imported into CytoHubba plugin, which helped to identify key genes through 5 different calculation methods, namely, EPC, MCC, DMNC, MNC, and Degree (**Table 3**). Subsequently, the data were imported into another plugin, MCODE, which helped to identify different clusters. The results showed that MT1G, MT1X, MT1F, MT1H and MT1M were in cluster 1 while FABP4, SPP1, MMP7 and CCL2 were in cluster 2 (cutoff k-score = 2). Finally, we performed a correlation analysis of these 9 core genes (MT1G, MT1X, MT1F, MT1H, MT1M, FABP4, SPP1, MMP7 and CCL2) and found that they were closely related to each other (**Figure 5B**).

**Table 3:** Genes filtered by Cytoscape software (CytoHubba plugin and MCODE plugin).

NAME	MCC RANK	DMNC RANK	MNC RANK	Degree RANK	EPC RANK	Total Rank
MT1G	1	1	1	3	7	1
MT1H	2	2	2	4	8	2
CCL2	6	7	7	1	1	3
SPP1	7	6	5	2	2	4
MT1F	3	3	3	6	9	5
MT1M	4	4	4	7	11	6
MT1X	5	5	6	9	10	7
MMP7	10	8	8	10	5	8
FABP4	12	9	9	12	4	9
IL7R	9	15	15	8	3	10
CFTR	8	16	16	5	6	11
GPC3	11	13	13	11	12	12
CXCL6	14	10	10	14	13	13
CLIC6	15	11	11	15	17	14
KRT7	16	12	12	16	18	15
HKDC1	13	18	18	13	14	16
CD24	17	14	14	17	15	17
AKR1B10	18	17	17	18	19	18
SLC12A2	19	19	19	19	16	19

▀▀▀ MOCDE1.

▀▀▀ MCODE2.

### Construction of LASSO Logistic Regression Classifiers

To develop classifiers to distinguish different NAFLD states, we performed LASSO logistic regression based on the expression of the 9 core genes (MT1G, MT1X, MT1F, MT1H, MT1M, FABP4, SPP1, MMP7 and CCL2). Our calculation followed the 10-fold cross-validation method and randomly divided the patients into groups- training group: testing group = 6:4.

For Normal-NASH group, 6 genes (CCL2, FABP4, MT1G, MT1H, MT1M, and SPP1) were identified as optimal features by non-zero regression coefficients (**Figure 5C**). The classifier’s lambda.min = 0.107641 and the mean squared errors (MSE) = 0.082318791. Later, a gene-based classifier index was created with the following formula: Index=CCL2*(-0.225381325) + FABP4*(1.739726652) + MT1G*(0.78448675) + MT1H * (0.451076017) + MT1M*(-1.083839703) + SPP1*(1.009899707) + (-21.10137666). **Figure 5D** presented the results of PCA before feature selection and **Figure 5E** presented the results of PCA with genes identified by LASSO methods, which indicated that samples with different NAFLD states (Normal/NASH) are more easily distinguished by Normal-NASH classifier. The accuracy of the Normal-NASH classifier was 0.8878 in the training group, 0.9219 in the testing group, and 0.9012 in the total group. Based on the accuracy, Se, Sp, PPV, NPV, and AUC values showed that sample recognition efficiency of the classifier was high (**Table 4**). The ROC curve showed that AUC was 0.944 in the training set, 0.965 in the testing group, and their difference was not significant (Delong method (23) P = 0.5125, **Figure 5F**).

**Table 4:** Validation of LASSO logistic regression classifiers.

NAME	Group	SE	SP	PPV	NPV	Accuracy	AUC
Normal-NASH	Train	0.814	0.9455	0.9211	0.8667	0.8878	0.9438
	Test	0.8636	0.9524	0.9048	0.9302	0.9219	0.9654
	Total	0.8308	0.9485	0.9153	0.8932	0.9012	0.9526
NAFL-NASH	Train	0.7027	0.825	0.7879	0.75	0.7662	0.9149
	Test	0.8214	0.6957	0.7667	0.7619	0.7647	0.9146
	Total	0.7538	0.7778	0.7778	0.7538	0.7656	0.9104
Normal-NAFL	Train	0.4242	0.8889	0.6667	0.7467	0.7292	0.7201
	Test	0.5333	0.8529	0.7619	0.6744	0.7031	0.7304
	Total	0.4762	0.8763	0.7143	0.7203	0.7188	0.73

AUC, area under the receiver operating characteristic curve; NPV, negative predictive value; PPV, positive predictive value; Se, sensitivity; Sp, specificity.

For Normal-NAFL group, 9 genes (MT1G, MT1X, MT1F, MT1H, MT1M, FABP4, SPP1, MMP7, and CCL2) were identified as optimal features by non-zero regression coefficients (**Figure 5G**). The classifier was lambda.min = 0.001001753 and MSE = 0.197118182. Later, a gene-based classifier index was created with the following formula: Index=CCL2*(-0.190052713) + FABP4 * (0.675427091) +MMP7*(-0.91795346) +MT1X *(-0.181266023) + MT1F*(-0.777987595) + MT1G* (0.135941039) + MT1H * (1.336138959) + MT1M*(-0.42412614) + SPP1 * (0.605956212) + (-1.012626846). **Figure 5H** presented the results of PCA before feature selection and **Figure 5I** presented the results of PCA with genes identified by LASSO methods, which indicated that samples with different NAFLD states (Normal-NAFL) are more easily distinguished by Normal-NAFL classifier. The accuracy of the Normal-NAFL classifier was 0.7292 in the training group, 0.7031 in the testing group, and 0.7188 in the total group. Based on the accuracy, Se, Sp, PPV, NPV, and AUC values showed that sample recognition efficiency of the classifier was high (**Table 4**). The ROC curve showed that the AUC was 0.720 in the training set, 0.730 in the testing group, and their difference was not significant (Delong method (23) P = 0.9051, **Figure 5J**).

For the NAFL-NASH group, 8 genes (MT1G, MT1F, MT1H, MT1M, FABP4, SPP1, MMP7, and CCL2) were identified as optimal features with non-zero regression coefficients (**Figure 5K**). The classifier was lambda.min = 0.006254024 and MSE = 0.123431321. Later, a gene-based classifier index was created with the following formula: Index=CCL2*(-0.166311656) + FABP4 * (0.494621105) +MMP7*(1.434246184) + MT1F*(2.35905055) + MT1G*(1.159066248) + MT1H * (-2.090099881) + MT1M*(-0.843261703) + SPP1*(0.442308223) + (-29.40911129). **Figure 5L** presented the results of PCA before feature selection and **Figure 5M** presented the results of PCA with genes identified by LASSO methods, which indicated that samples with different NAFLD states (NAFL/NASH) are more easily distinguished by NAFL-NASH classifier. The accuracy of the NAFL-NASH classifier was 0.7662 in the training group, 0.7647 in the testing group, and 0.7656 in the total group. Based on the accuracy, Se, Sp, PPV, NPV, and AUC values showed that the sample recognition efficiency of the classifier was high (**Table 4**). The ROC curve showed that the AUC was 0.915 in the training group, 0.915 in the testing group, and their difference was not significant (Delong method (23) P = 0.9956, **Figure 5N**).

Besides, many studies showed that PNPLA3 is one of the genetic risk factors with more evidence in the NAFLD progression (7, 24, 25), so we performed ROC curves to compare the ability of PNPLA3/classifiers to distinguish different NAFLD states. The results showed that classifiers have better accuracy and reliability (AUC=0.953, 0.730, 0.910) than PNPLA3 (AUC=0.579, 0.639, 0.565) to distinguish NAFLD states (Normal/NAFL/NASH) (**Figures 5O–Q**).

### Correlation of the Classifier Index and Clinical Characteristics

**Figure 6:**
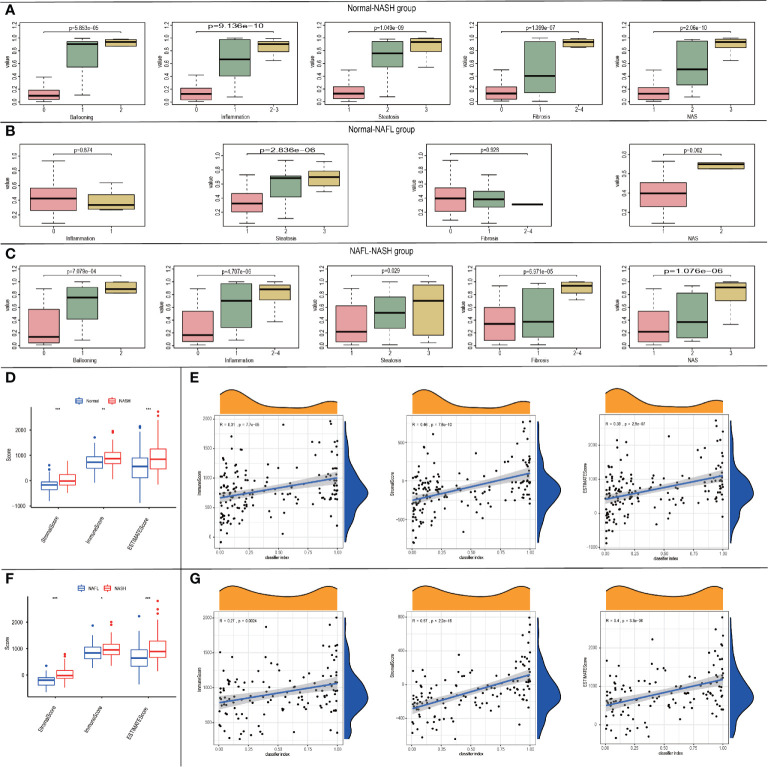
The relationship between the classifier index and clinical characteristics. **(A-C)** Boxplots of the relationship between classifier index and clinical characteristics in Normal-NASH group **(A)**, Normal-NAFL group **(B)**, NAFL-NASH group **(C)**; **(D)**Boxplot of the relationship between classifier scores and immune microenvironment scores in Normal-NASH group; **(E)** Diagram validating correlation between classifier scores and immune microenvironment scores in Normal-NASH group; **(F)** Boxplot of the relationship between classifier scores and immune microenvironment scores in NAFL-NASH group; **(G)** Diagram validating correlation between classifier scores and immune microenvironment scores in NAFL-NASH group.

Considering the correlation among classifiers and NAFLD states, we tried to explore the relationships between classifier index and clinical characteristics. In the Normal-NASH group, the classifier index was significantly associated with ballooning grade (p=5.853e^-05^), inflammation (p=9.136e^-10^), steatosis (p=1.049e^-09^), fibrosis (p=1.399e^-07^) and NAS (p=2.06e^-10^). As the classifier index increased, the level of ballooning/inflammation/steatosis/fibrosis/NAS elevated (**Figure 6A**). In Normal-NAFL group, classifier index was significantly associated with steatosis (p=2.836e^-06^) and NAS (p=0.002). However, classifier index showed no significant relationship with ballooning/fibrosis. As the classifier index increased, the level of steatosis/NAS elevated (**Figure 6B**). In NAFL-NASH group, results showed classifier index was significantly associated with ballooning (p=7.079e^-04^), inflammation (p=4.707e^-06^), steatosis (p=0.029), fibrosis (p=6.971e^-05^), and NAS (p=1.076e^-06^) (**Figure 6C**). Meanwhile, the level of ballooning/inflammation/steatosis/fibrosis/NAS elevated as classifier index increased. The above results showed that our classifier can also be used to predict the NAFLD patients with different clinical characteristics.

### Relationship Between Classifiers and Immune Score

To explore the relationship between the classifier and immune microenvironment, we performed correlation analysis among the classifier index and three indexes (StromalScore, ImmuneScore and ESTIMATEScore, which were acquired from “estimate” package in R software). In the Normal-NASH group, StromalScore, ImmuneScore, and ESTIMATEScore were higher than those of the normal group (**Figure 6D**). Besides, classifier index showed a medium correlation with StromalScore (Pearson R = 0.46, p = 7.6e^-10^), ImmuneScore (Pearson R = 0.31, p = 7.7e^-05^) and ESTIMATEScore (Pearson R = 0.39, p = 2.9e^-07^) (**Figure 6E**). There was no difference in StromalScore, ImmuneScore and ESTIMATEScore in the Normal-NAFL group. In the NAFL-NASH group, StromalScore, ImmuneScore, and ESTIMATEScore of the NASH group were higher than those of the NAFL group (**Figure 6F**), and the classifier index showed a high correlation with StromalScore (Pearson R = 0.57, p < 2.2e^-16^), low correlation with ImmuneScore (Pearson R = 0.27, p = 0.0024) and medium correlation with ESTIMATEScore (Pearson R = 0.4, p = 3.3e^-06^) (**Figure 6G**).

Therefore, our classifier can also be used to predict the immune microenvironment of patients with different disease states.

### Correlation Between Progress-Related DEGs and Clinicopathological Traits

**Figure 7:**
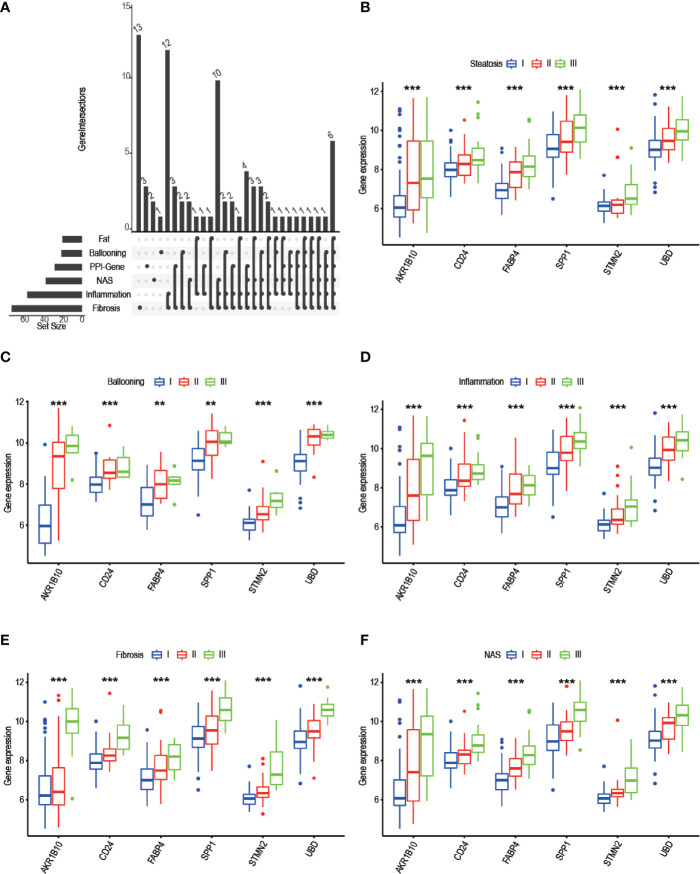
Screening of progress-related genes. **(A)** UpSet plot including trait related genes and PPI-related genes; **(B-F)** 6 progress-related genes’ expression in different degree of steatosis **(B)**, ballooning **(C)**, inflammation **(D)**, fibrosis **(E)**, NAS **(F)**. (* P< 0.05, ** P< 0.01, *** P< 0.001).

To identify progress-related genes, we performed an Upset plot among clinicopathological-related DEGs (ballooning, steatosis, inflammation, fibrosis, NAS) and 19 filtered genes obtained from the STRING database. We found 6 intersected genes, namely, AKR1B10, SPP1, CD24, UBD, FABP4, and STMN2 (**Figure 7A**). Subsequently, we explored the relationship between AKR1B10/SPP1/CD24/UBD/FABP4/STMN2 expression and clinicopathological characteristics. As shown in pictures, the above gene expressions were remarkably correlated with steatosis (**Figure 7B**), ballooning (**Figure 7C**), inflammation (**Figure 7D**), fibrosis (**Figure 7E**), and NAS (**Figure 7F**). As the level of clinical characteristics increase, these genes expressions also increase. Therefore, we defined these 6 genes (AKR1B10/SPP1/CD24/UBD/FABP4/STMN2) as progress-related genes that play a vital role in the progression of NAFLD.

### Correlation of Six Progress-Related Genes With HCC

**Figure 8:**
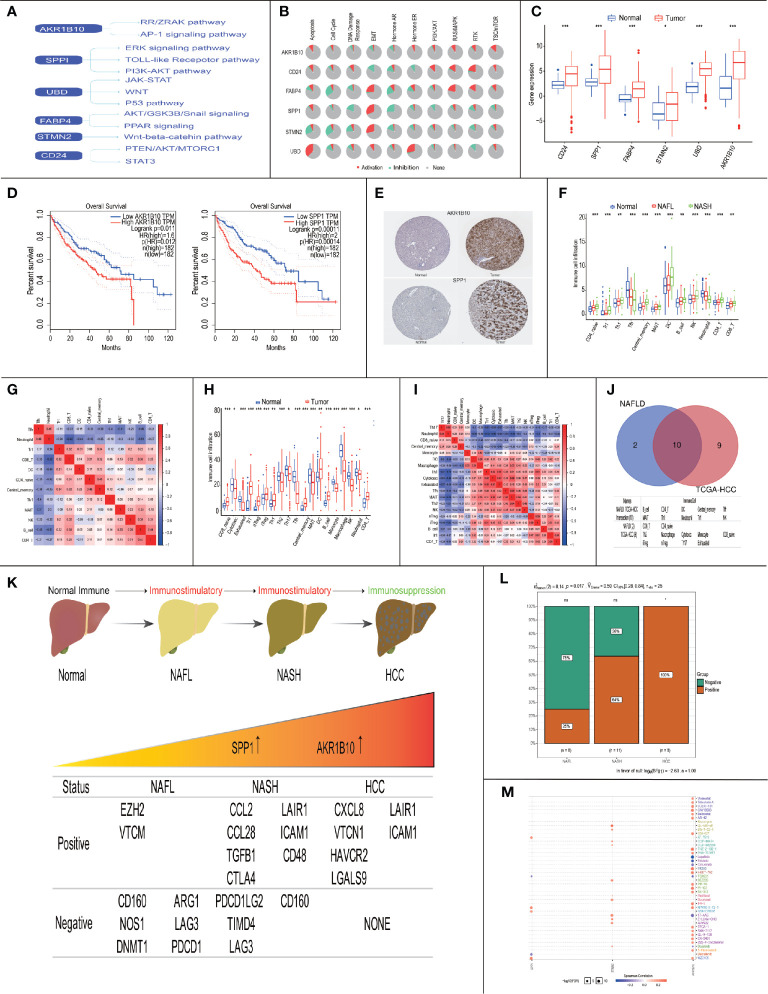
Immune landscape and treatment prediction in NAFLD patients and HCC patients. (* P< 0.05, ** P< 0.01, *** P< 0.001). **(A)** Literature-based of six progress-related genes in HCC-associated signaling pathways; **(B)** Enriched pathways of 6 progress-related genes in TCGA database; **(C)** Expression of progress-related genes between normal patients and HCC patients in the TCGA database; **(D)** Survival analysis of AKR1B10 and SPP1; **(E)** Protein expression of AKR1B10 and SPP1 between normal patients and HCC patients; **(F)** Boxplots visualizing the difference of immune cell infiltration in Normal/NAFL/NASH patients; **(G)** Correlation analysis of immune cells in Normal/NAFL/NASH patients; **(H)** Boxplots visualizing the difference of immune cell infiltration in normal/HCC patients; **(I)** Correlation analysis of immune cells in Normal/HCC patients; **(J)** The intersection of immune cells between NAFLD patients and HCC patients; **(K)** The relationship between immunosuppressive cytokines expression and AKR1B10/SPP1; **(L)** Chi-square test of immunosuppressive cytokines expression in the progress of NAFLD; (M) Drug sensitivity analysis of AKR1B10 and SPP1.

Previous studies showed that up to one-third of patients with NASH might progress to HCC (26, 27), so we explored the role of six progress-related genes (AKR1B10/SPP1/CD24/UBD/FABP4/STMN2) in HCC. In previous research, we found that these progress-related genes were closely related to tumorigenesis and HCC progression (**Figure 8A**). Moreover, based on the TCGA database, we found that progress-related genes were enriched in tumor-related pathways, such as apoptosis, cell cycle, and EMT (**Figure 8B**).

In the TCGA database, we found that six progress-related genes were up-regulated in the HCC (**Figure 8C**). To explore the relationship between six progress-related genes and prognosis, we performed survival analysis and the results showed that high expression of SPP1 (p=0.00011) and AKR1B10 (p=0.011) were associated with a low survival rate (**Figure 8D**). In HPA database, the expression of SPP1/AKR1B10 was also abnormally elevated in HCC (**Figure 8E**). Therefore, SPP1/AKR1B10 may be closely related to progress and prognosis in Normal-NAFL-NASH-HCC progression.

### The Immune Landscape of NAFLD and HCC Patients

Subsequently, we explored the immune landscape of NAFLD and HCC. In NAFLD, the results showed that the immune infiltration of the following cells increased (CD4_naïve cells, Tr1 cells, Th1 cells, Central memory cells, Dendritic Cells (DCs), B cells, NK cells, CD4 T cells, and CD8 T cells), while the immune infiltration of Tfh cells and Neutrophil decreased (**Figure 8F**). To investigate the underlying relationships among these immune cells, we evaluated the correlation between each other. We found that Neutrophils and Tfh cells appeared to have the most positive correlation (R = 0.46), while Neutrophils and CD8+ T cells had the most negative correlation (R = -0.62) (**Figure 8G**). The above results indicated that in the course of Normal-NAFL-NASH, the immune microenvironment was gradually activated and reached its peak at the time of NASH, which was consistent with the conclusion that NASH is an inflammatory disease (28).

In HCC, the immune infiltration of the following cells increased (CD8 naive cells, Tr1 cells, nTreg cells, iTreg cells, Th1 cells, Tfh cells, Central memory cells, DC cells, B cells, and CD4 T cells), while the immune infiltration of these cells decreased (Cytotoxic cells, Exhausted T cells, Th2 cells, Th17 cells, MAIT cells, Monocyte, Macrophage, NK cells, and Neutrophil)(**Figure 8H**). Besides, we found that Exhausted T cells and Cytotoxic cells appeared to have the most positive correlation (R = 0.67), while Neutrophils and Th1 cells had the most negative correlation (R = -0.72) (**Figure 8I**). These results showed that the immune microenvironment of HCC was in a suppressed state.

Given that intersected immune cells between NAFLD and HCC may affect the progression and prognosis of the disease, we performed survival analysis and found that Cytotoxic cells (p=0.007), MAIT cells (p=0.017), Tfh cells (p<0.001), Th2 cells(p=0.004), B cells (p=0.006), and DCs (p=0.034) were closely related to survival (**Figures 8J**, **S1A**).

Therefore, these results indicated that immune status changed from immune-activation to immune-suppression in the process of NASH to HCC. Besides, our study may provide therapeutic targets (Cytotoxic cells, MAIT cells, Tfh cells, Th2 cells, B cells and DCs) for NAFLD to slow down progression and improve prognosis.

### Correlation of AKR1B10/SPP1 With Immune Microenvironment

Subsequently, we explored the relationship between prognosis related genes (SPP1/AKR1B10) and survival-related immune cells (Cytotoxic cells, MAIT cells, Tfh cells, Th2 cells, B cells, and DCs). The results showed that the expression of AKR1B10 was positively correlated with the content of DCs (R = 0.18, p = 0.00039) and MAIT cells (R = 0.22, p = 1.7e-05), the expression of SPP1 was also positively correlated with the content of DCs (R = 0.39, p = 5.3e-15) and MAIT cells (R = 0.21, p = 6e-05) (**Figure S1B**).

Later, we explored the relationship between NAFLD progression and immunosuppressive cytokines’ expressions. Based on previous research, we downloaded Cancer-Immunity Cycle associated immunosuppressive cytokines from the Tracking Tumor Immunophenotype website (29). We defined p<0.05 and R≥0.3 as the threshold to screen out co-expressed immunosuppressive cytokines of SPP1/AKR1B10 (**Figures S1C-E**). The results showed that the ratio of genes involving the negative regulation of the Cancer-Immunity Cycle increased, indicating activities of the Cancer-Immunity Cycle gradually decreased in progress of NAFL-NASH-HCC (**Figures 8K, L**).

The above results indicated that SPP1/AKR1B10 might play a vital role in the change of the immune microenvironment. Besides, based on the GDSC database, we performed a drug sensitivity analysis on six progress-related genes. It found that TGX-221 was a common sensitive drug of AKR1B10 and SPP1, which may play a role in inhibiting or delaying the progression of NAFLD and improve the prognosis of NAFLD-HCC (**Figure 8M**).

## Discussion

Non-alcoholic Fatty Liver disease (NAFLD) is currently considered as the first cause of chronic liver disease accounting for 25% of cases worldwide (4). NAFLD is generally divided into two stages: the early stage is non-alcoholic fatty liver (NAFL) with pathological features of isolated steatosis, no or minimal inflammatory activity, and no evidence of cell damage. In the progress of NAFL to non-alcoholic steatohepatitis (NASH), namely second stage, inflammation and liver cell damage characterized by hepatocyte swelling appear in the liver, accompanied by various degrees of fibrosis (30, 31). Lipid accumulation, liver cell damage, immune system dysfunction and fibrosis are all involved in NAFLD, which may eventually progress to HCC (32). Early detection and diagnosis are of great importance in NAFLD treatment. Up to now, NAFLD diagnosis mainly relies on imaging examination and liver biopsy, which lacks ability to precisely assess disease status and predict NAFLD progression.

Based on 111 intersected DEGs between Normal-NASH group and NAFL-NASH group, we performed GO and KEGG pathway analysis to explore underlying mechanism of NAFLD. The results showed that enriched pathways were involved in tumorigenesis, such as extracellular matrix organization, extracellular structure organization and PI3K-AKT signaling pathway. Meanwhile, most overlapping pathways in GSEA were also related to tumorigenesis among Normal-NASH group and NAFL-NASH group, such as EMT, angiogenesis and p53 pathway (**Figure 2**, **3**). These results indicated that extracellular matrix may play an important role in the development of NAFLD

Subsequently, we constructed a PPI network of NAFLD. Based on specific gene functions, we divided PPI network into four modules: Ballooning Associated, Inflammation Associated, Fibrosis Associated, and Glycolysis Associated. This was roughly the same as the development process of NAFLD (26, 27). Although patients in “first-hit” can reverse histological steatosis to a normal state, when progressing to NASH (second-hit), patients have been in an irreversible state. Thus, early detection and timely treatment are of great importance to stop and reverse NAFLD progression. So far, the diagnosis of the NAFLD state still mainly depends on pathology, whereas it is expensive and inconvenient to operate (33–35). In past years, some models, such as hepatic-portal venous pressure gradient (HVPG), computed tomography (CT), MRI, and MR elastography (MRE), were built to diagnose NAFLD disease state (36). However, low sensitivity and high false-positive rates limit their clinical use. Given the important link between genes and NAFLD progression (37), proper gene signature classifiers may provide simple and accurate evaluation methods for NAFLD status.

Here, based on nine core-gene expressions (MT1G, MT1X, MT1F, MT1H, MT1M, FABP4, SPP1, MMP7 and CCL2) filtered by WCGNA and Cytoscape, we developed three classifiers to successfully identify different NAFLD states (sensitivity Normal/NAFLD (47%), NAFLD/NASH (75%), Norma/NASH (83%) and specificity Normal/NAFLD (87%), NAFLD/NASH (77%), Normal/NASH (94%), **Figure 4**, **5**). Whereas compared with serum aspartate aminotransferase (AST), previous studies showed suboptimal diagnostic utility (sensitivity 42% and specificity 80% using ALT > 30U/L as a cutoff) in diagnosing Normal/NASH (36). Our classifiers seemed to show limited sensitivity in distinguishing Normal/NAFLD, which might be attributed to few gene expression changes between simply steatosis and normal liver, as liver is physiologically the organ of lipids accumulation and metabolism. Along with these results, three gene-based classifiers could feasibly and robustly predict NAFLD states from a biological perspective.

Later, we proved classifiers could effectively distinguish clinical characteristics (ballooning, inflammation, steatosis, fibrosis and NAS) in Normal/NASH and NAFLD/NASH group. In Normal/NAFL group, the classifier score was associated with steatosis and NAS, while no inflammation and fibrosis may be due to little inflammation and fibrosis occurring in patients in first-hit. Meanwhile, the classifier score was closely associated with an immune microenvironment score in Normal-NASH/NAFL-NASH, which indicated classifiers could also predict states of the immune microenvironment (**Figure 6**).

To clarify the relationship between genes and NAFLD progress, we performed an upset-plot between pathological-related DEGs and 19 genes obtained from STRING database. Six progress-related genes (AKR1B10/SPP1/CD24/UBD/FABP4/STMN2) were found remarkably correlated with steatosis, ballooning, inflammation, fibrosis, and NAS in the progress of Normal/NAFL/NASH. Subsequently, we explored the relationship between these six progress-related genes and NAFLD-HCC prognosis. Previous studies showed that these six progress-related genes play a vital role in tumorigenesis, such as PI3K-AKT pathway (38),p53 pathway (39), and STAT pathway (40). Meanwhile, in the TCGA database, the six progress-related gene expressions were increased in HCC patients, and SPP1/AKR1B10 was negatively related to overall survival. AKR1B10 was reported to metabolize a variety of substrates, such as retinal, lipid peroxidation products, and exogenous biological agents (41–44). As storage of retinyl in cytoplasmic lipid droplets is the most distinctive feature of hepatic stellate cell (45), therefore, the abnormal expression of AKR1B10 may lead to the activation of HSC and promote the progression of NAFLD (46). Furthermore, studies have demonstrated that AKR1B10 was also related to tumor growth and metastasis, which may explain the lasting role in NAFL-NASH-HCC progression (47–49). Secreted phosphoprotein 1 (SPP1), also known as osteopontin (OPN), is a secreted glycoprotein that has multiple functions and affects proliferation, differentiation, migration and inflammation (50–52). Abnormal SPP1 expression was related to various cancer progressions [colorectal cancer (53, 54), lung cancer (55), and HCC (56)] *via* inducing inflammation and reshaping the microenvironment. As SPP1 and AKR1B10 were closely related to the Norma-NAFL-NASH process and the prognosis of HCC, these two genes may be key genes in the progression of Normal-NAFL-NASH-HCC.

As deregulated immune microenvironment was proved to have a profound effect on the progression of NAFLD progression *via* inflammation, we tried to explore immune microenvironment changes in Normal-NAFL-NASH-HCC. The results showed that immune activated cells (CD4 T, CD8 T and NK) infiltrations gradually increased, indicating an immunostimulatory microenvironment remodeling during Normal-NAFL-NASH progress. On the contrary, immune activated cell infiltration decreased and immunosuppressive cell infiltrations gradually increased in HCC, indicating a suppressive immune microenvironment. The deregulated ratio of immune cells and cytokines reshaped microenvironment, which may be a key factor in the conversion of NAFL/NASH/HCC (57–59). Therefore, we performed a survival analysis of immune cells to look for prognosis-related immune cells and six immune cells (Cytotoxic cells, MAIT cells, Tfh cells, Th2 cells, B cells and DCs) were identified as being survival-related.

Later, we explored the relationship between AKR1B10/SPP1 and six immune cells. The results showed that AKR1B10 was positively related to DCs and MAIT cells. And SPP1 was positively related to B cells, DCs, and MAIT cells. In B cells, which account for half of the total number of lymphocytes in the liver, the infiltration gradually increases during the progression of NAFLD. Previous studies showed CCl4-induced B-cell deficient mice have reduced liver fibrosis, which indicated that B cells had the pro-fibrotic capability (60, 61). As mentioned earlier, the key character of NASH was the loss of the liver’s tolerance to the microenvironment, which turned the liver into a pro-inflammatory immune phenotype and subsequently released pro-inflammatory cytokines to induce DCs maturation, and finally increased adaptive immune responses by CD4+/CD8+ T cells activation and infiltration (62, 63). Therefore, DCs serve as a central cell bridge connecting the innate immune system and adaptive immune system responses, which work as antigen-presenting cells in the liver. Besides, consuming CD11c + DCs or CD103 + DCs reduced proinflammatory cytokine and liver fibrosis in MCD-induced NASH or thioacetamide-diet–induced liver fibrosis models proved DCs also played a pro-inflammatory role in the NAFLD process (28, 64, 65).

Human mucosal-associated invariant T cells (MAIT cells) are highly enriched in liver and highly conservative at the evolutionary level. They express semi-constant T cell receptors (TCR), which can specifically recognize microbial-derived vitamin B metabolites, and then release a large number of inflammatory cytokines and granzymes (66, 67). The role of MAIT cells in NASH process is not yet clear. Previous studies showed that MAIT cells increase during acute injury or infection, which promoted inflammation and protected the body (68). However, when disease progresses or turns into a chronic disease, MAIT cells start to decrease. Meanwhile, the gene expression pattern of MAIT cells will change. MAIT cells in Autoimmune liver disease (AILD) patients had evolved into an exhausted, pro-fibrotic phenotype, and promoted the development of HSC-mediated liver fibrosis (69, 70). Compared to normal samples, although MAIT cells reduced in HCC, tumor-derived MAIT cells down-regulated genes enriched in the cytokine secretion and cytolytic effect pathways (NFKB1 and STAT5B) and up-regulated genes such as IL8, CXCL12 and HAVCR2 (TIM -3) promote tumor development (71). Therefore, MAIT cells may harm the individual’s immune defense in chronic diseases, especially in NAFLD/HCC. The correlation between AKR1B10, SPP1, B cells, DCs, and MAIT cells indicated the possible interactive network structure that may explain and control metabolic disorders and fibrotic phenotypes from NASH to HCC. Therefore, we selected AKR1B10 and SPP1 co-sensitive drugs TGX-221 as a possible therapy to inhibit or delay the progression of NAFLD and improve the prognosis of NAFLD-HCC.

## Conclusion

In conclusion, we established 3 gene-based signature classifiers that may serve as biomarkers to predict disease state in NAFLD. In our analysis, we also discovered changes in the immune microenvironment, the key immune cells (B cells, DCs, MAIT cells) and genes (AKR1B10, SPP1) in the progression of NAFLD. Besides, TGX-221 may be a potential therapeutic drug for the treatment of NAFLD and NAFLD-HCC.

## Data Availability Statement

The original contributions presented in the study are included in the article/**Supplementary Material**. Further inquiries can be directed to the corresponding authors.

## Author Contributions

JW conceptualized the discussions. FZ wrote the manuscript under the supervision of YG. FZ, YZ, XH, and MZ extracted, analyzed data, and edited the manuscript. All authors contributed to the article and approved the submitted version.

## Funding

This work was supported by the National Key R&D Program of China(2018YFC1106400; 2018YFA0108200); Science and Technology Planning Project of Guangdong Province(2015B020229002), the National Natural Science Foundation of China (31972926), The Natural Science Foundation of Guangdong Province (2014A030312013,2018A030313128); Guangdong key research and development plan (2019B020234003); Science and Technology Program of Guangzhou (201803010086).

## Conflict of Interest

The authors declare that the research was conducted in the absence of any commercial or financial relationships that could be construed as a potential conflict of interest.

## Supplementary Material

Supplementary Figure 1 Immune landscape in NAFLD patients and HCC patients. **(A)** Survival analysis of immune cells in HCC patients; **(B)** Relationship of AKR1B10/SPP1 and survival-related immune cells; **(C–E)** Relationship between the AKR1B10/SPP1 and immunosuppressive cytokines in NAFL group **(C)**; NASH group **(D)**; HCC group** (E)**.
