# Supplementary figures and images for: Predicting Non-Alcoholic Fatty Liver Disease Progression and Immune Deregulations by Specific Gene Expression Patterns
Source: Front Immunol. 2021 Jan 26;11:609900. doi: 10.3389/fimmu.2020.609900 (PMC7870871; doi:10.3389/fimmu.2020.609900)

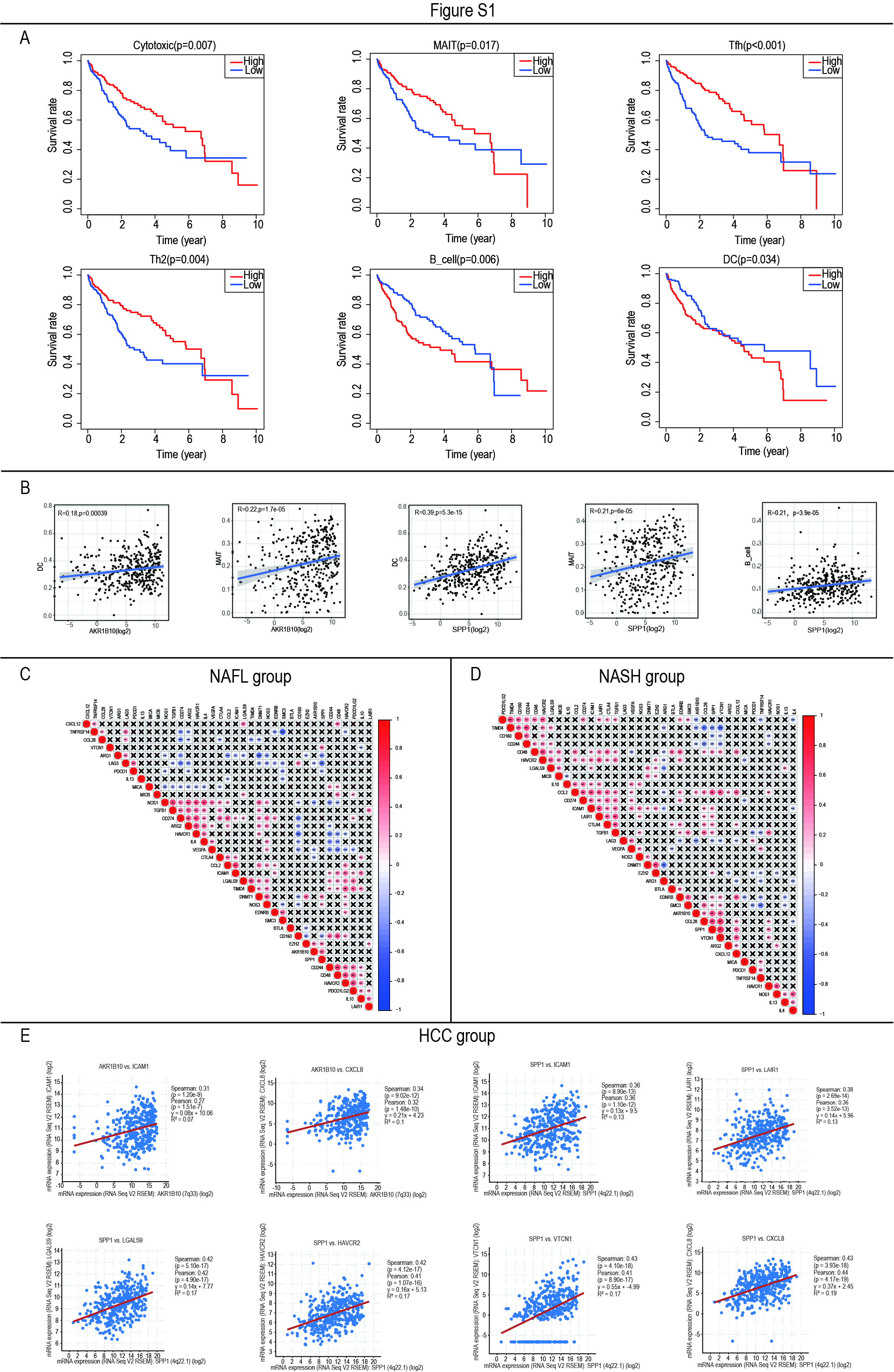

Supplement: Supplementary Figure 1 — Immune landscape in NAFLD patients and HCC patients. (A) Survival analysis of immune cells in HCC patients; (B) Relationship of AKR1B10/SPP1 and survival-related immune cells; (C–E) Relationship between the AKR1B10/SPP1 and immunosuppressive cytokines in NAFL group (C); NASH group (D); HCC group (E). [file Image_1.tif]
